# In Silico Core Proteomics and Molecular Docking Approaches for the Identification of Novel Inhibitors against *Streptococcus pyogenes*

**DOI:** 10.3390/ijerph182111355

**Published:** 2021-10-28

**Authors:** Abdur Rehman, Xiukang Wang, Sajjad Ahmad, Farah Shahid, Sidra Aslam, Usman Ali Ashfaq, Faris Alrumaihi, Muhammad Qasim, Abeer Hashem, Amal A. Al-Hazzani, Elsayed Fathi Abd_Allah

**Affiliations:** 1 Department of Bioinformatics and Biotechnology, Government College University Faisalabad, Faisalabad 38000, Pakistan; abdurrehman93@gcuf.edu.pk (A.R.); farahshahid24@gcuf.edu.pk (F.S.); qasemawan@gmail.com (M.Q.); 2 College of Plant Science and Technology, Huazhong Agricultural University, Wuhan 430070, China; wangxiukang@yau.edu.cn; 3 Department of Health and Biological Sciences, Abasyn University, Peshawar 25000, Pakistan; sajjad.ahmad@abasyn.edu.pk; 4 Department of Medical Laboratories, College of Applied Medical Sciences, Qassim University, Buraydah 51452, Saudi Arabia; f_alrumaihi@qu.edu.sa; 5 Botany and Microbiology Department, College of Science, King Saud University, P.O. Box 2460, Riyadh 11451, Saudi Arabia; habeer@ksu.edu.sa (A.H.); alhazzani@ksu.edu.sa (A.A.A.-H.); 6 Plant Production Department, College Food and Agricultural Sciences, King Saud University, P.O. Box 2460, Riyadh 11451, Saudi Arabia; eabdallah@ksu.edu.sa

**Keywords:** *Streptococcus pyogenes*, multidrug resistance, cross-resistance, phytochemicals, inhibitors, pathogenic

## Abstract

*Streptococcus pyogenes* is a significant pathogen that causes skin and upper respiratory tract infections and non-suppurative complications, such as acute rheumatic fever and post-strep glomerulonephritis. Multidrug resistance has emerged in *S. pyogenes* strains, making them more dangerous and pathogenic. Hence, it is necessary to identify and develop therapeutic methods that would present novel approaches to *S. pyogenes* infections. In the current study, a subtractive proteomics approach was employed to core proteomes of four strains of *S. pyogenes* using several bioinformatic software tools and servers. The core proteome consists of 1324 proteins, and 302 essential proteins were predicted from them. These essential proteins were analyzed using BLASTp against human proteome, and the number of potential targets was reduced to 145. Based on subcellular localization prediction, 46 proteins with cytoplasmic localization were chosen for metabolic pathway analysis. Only two cytoplasmic proteins, i.e., chromosomal replication initiator protein DnaA and two-component response regulator (TCR), were discovered to have the potential to be novel drug target candidates. Three-dimensional (3D) structure prediction of target proteins was carried out via the Swiss Model server. Molecular docking approach was employed to screen the library of 1000 phytochemicals against the interacting residues of the target proteins through the MOE software. Further, the docking studies were validated by running molecular dynamics simulation and highly popular binding free energy approaches of MM-GBSA and MM-PBSA. The findings revealed a promising candidate as a novel target against *S. pyogenes* infections.

## 1. Introduction

*S. pyogenes* is a catalase-negative, β-hemolytic, oxidase-negative, and Gram-positive streptococcus. It is also called group A streptococcus (GAS). On blood agar plates, it forms pinpoint colonies at 5 to 10% CO_2_ [1]. *S. pyogenes* cells are non-motile and generally grow in the form of pairs or chains [2]. *S. pyogenes* infections are extremely contagious. Airborne droplets, bacterially contaminated surfaces or objects, hand contact with nasal discharge, skin contact with contaminated lesions, or contaminated food sources are all possible modes of transmission. The frequency of *S. pyogenes* infections varies depending on the clinical manifestations of the infection in different parts of the world [3].

*S. pyogenes* is accountable for a variety of diseases, such as pharyngitis, scarlet fever, erysipelas, necrotizing fasciitis, septicemia, cellulitis, acute glomerulonephritis, rheumatic fever, and toxic shock syndrome [4]. *S. pyogenes* infections in humans have a significant economic impact, but no potential drug exists to prevent them [5].

Intramuscular benzathine or oral penicillin for 10 days is the preferred treatment for bacterial pharyngitis. This is a low-cost treatment with a limited range of activities. Clindamycin or vancomycin can be used to treat severe invasive *S. pyogenes* infections [6,7]. The macrolides are now considered a third-line treatment for streptococcal throat infections because few strains of *S. pyogenes* have become resistant to them [8]. The emergence of antibiotic-resistant Streptococci strains has shifted the focus of medical research toward the progress of new vaccines and novel antibiotics [9].

Hence, finding new targets in *S. pyogenes* is important. Various genomic disciplines can be combined to discover new pharmacological targets for inhibitor development. The likelihood of identifying relevant targets by computational approaches and the integration of “omics” data, such as metabolomics, genomics, and proteomics, has steadily increased in the present postgenomic era. Proteomic techniques, i.e., comparative and subtractive proteomics, are increasingly commonly used for identifying and predicting potential drug targets for various pathogenic bacteria [10]. In comparison with traditional methods, these methods save time, are faster, and are more cost-effective in the drug design process [11]. Using the subtractive proteomics approach, potential targets and vaccine candidates for various pathogenic bacteria have been identified over the last several years [12,13]. 

In the current study, the core proteome of four strains of *S. pyogenes* was analyzed to employ various subtractive proteomics approaches. The essential proteins required for bacterial survival were identified using a variety of computational software tools. To prevent potential drug cross-reactivity with host and bacterial proteins, we analyzed both metabolic and host non-homology pathways, as well as bacterial protein involvement in several host metabolic processes. The study was expanded to model the 3D structures of the likely drug targets using the SWISS-MODEL to identify a selective and potent inhibitor using docking studies. The findings of this study could help in the development of effective drug targets for *S. pyogenes*.

## 2. Materials and Methods

### 2.1. Core Proteome Retrieval

Complete proteomes of four *S. pyogenes* strains were downloaded from UniProt (European Bioinformatics Institute (EMBL-EBI), Hinxton, UK) [14]. UniProt is a comprehensive database of protein sequences and functional annotations. Proteomes were subjected to OrthoFinder program (University of Oxford, Oxford, UK) without altering the default parameters [15]. As the name OrthoFinder program indicates, this program performs calculations based on BLAST searches. Therefore, internal scripts were developed for the identification of core genes and the description of genes expressed in all strains being studied.

### 2.2. Identification of Drug Targets

The key cellular function of microbes is performed by essential proteins. Essential proteins are thought to be critical for cell survival [16]. Essential proteins were obtained through Geptop 2.0 server (University of Electronic Science and Technology of China, Chengdu, China) at cutoff score of 0.24 [17]. A BLASTp analysis of essential proteins was performed against the human proteome to identify functional similarities. The query coverage of >70% and identity of ≤30% were used to define non-homologous proteins. Predicting protein subcellular localization is important for genome annotations and genome analyses in bacteria because these proteins can be used as targets or candidates for vaccines. The BUSCA server (Bologna Biocomputing Group, Bologna, Italy) was employed for prediction of subcellular localization of proteins. Bacterial metabolic pathway enzymes that are both essential and common were also analyzed to identify drug targets [18]. The KEGG database was used to compare metabolic pathways in *H. sapiens* and *S. pyogenes* [19]. The metabolic pathways that are unique to *S. pyogenes* and not present in humans were chosen. Hence, proteins with unique metabolic pathways were chosen for further research.

### 2.3. Structure Prediction

After complete analysis and retrieval of target proteins sequence, those proteins were subjected for structure prediction. SWISS-MODEL tool was used for the structure prediction [20]. SWISS-MODEL is a complete functional protein structure homology modeling server that was accessed by Expasy web server.

### 2.4. Structure Evaluation

Accurate evaluation of the 3D model is considered as one of the core elements of computational structure prediction [21]. Scientists are making groundbreaking discoveries in computational structural biology as a result of new sequencing technologies that have emerged in recent years. The emergence of rapidly endorsed and highly efficient approaches for structure evaluation has paved new ways to qualitatively estimate the protein structures. In this work, refined drug targets were further qualitatively estimated using 4 independent programs: PROCHECK (Molecular Biology Institute and the DOE-MBI Institute, University of California, Los Angeles) [22], Verify 3D (Molecular Biology Institute and the DOE-MBI Institute, University of California, Los Angeles) [23], ERRAT [24], and ProsA-web (University of Salzburg, Salzburg, Austria) [25]. 

### 2.5. Preparation of Target Proteins

The Molecular Operating Environment software, version 2018.01, was used for energy minimization by picking the MMFF94x force-field [26]. The resulting structure was refined utilizing the Protonate3D program to add partial charges at a temperature of 310 K and pH value of 7. The Site Finder tool present in the MOE software detected the active sites in the target proteins.

### 2.6. Library Preparation

Using in silico methods, a thousand known phytochemicals were picked from various databases, such as PubChem, MPD3 [27], and Zinc, to screen their possible inhibitory impact on DNaA and TCR proteins. According to the literature survey, the plant-based phytochemicals were chosen based on their pharmacological effects [28]. The phytochemicals chosen were mostly alkaloids and sterols. The MOE software was utilized to create a ready-to-dock library of the selected phytochemicals [26]. ChemDraw was used to draw the two-dimensional (2D) chemical structure of the selected ligands. Before using the MOE ligand database, the ligands were refined using Protonate3D and the energy was decreased.

### 2.7. Molecular Docking Analysis

The active pocket on the receptor protein molecule was identified using MOE. The MOE software was used for the molecular docking approach to screen the library of 1000 phytochemicals against the interacting residues of DNaA and TCR proteins. The MOE software verified the proper ligand conformation to build a minimum energy structure using the “Triangular Matcher” algorithm, which was then used as the default ligand insertion strategy [26]. Rescoring of simulated poses was performed using the London dG scoring function in MOE. After docking, phytochemicals with the top and best conformation were identified based on RMSD values and S-score binding affinity. The MOE LigX tool was used to visualize the best-docked complexes and interpret the 2D plots of ligand–receptor interactions. MOE was also used to provide three-dimensional images of protein–inhibitor complexes.

### 2.8. Evaluation of Inhibitors’ Druglikeness 

The examination of a drug candidate’s druglikeness properties is an important phase in the drug discovery process. Several physical and chemical parameters, such as molecular weight, hydrogen bond acceptors, octanol–water partition coefficient log P (miLogP), and hydrogen bond donors, were analyzed. The top-docked ligands’ druggability was assessed using the Molinspiration online tool (https://www.molinspiration.com (accessed on 8 May 2021)) [29].

### 2.9. ADMET Profiling

Top phytochemicals pharmacokinetic properties were further evaluated by the SwissADME and admetSAR tools (http://www.swissadme.ch/ accessed on 9 May 2021)) [30,31]. Pharmacokinetic properties include toxicity, metabolism, absorption, and distribution in the human body. 

### 2.10. Molecular Dynamics Simulation Protocol

For dynamics understanding, MD simulations of the top two hits for both targets DNaA and TCR were performed. The AMBER20 software was used to conduct the simulation studies [32]. The antechamber program was used in the preprocessing of DNaA and TCR. For both targets, the GAFF force field was used, while for the enzyme, the ff14SB force field was used [33]. LEaP was used to record the topology of enzymes and inhibitors, counterions were introduced to bring electrostatic neutrality to the systems. The systems were housed in a water-molecule-filled TIP3P box [34]. The steepest descent approach was used for 1500 steps. The conjugate gradient method was then used for 1000 steps to get the lowest energy possible for the systems. A cutoff of 8 was set for non-bonding interactions. For ten minutes, the systems were heated to a constant temperature of 300 K and kept at a constant volume. The systems were then equilibrated for 100 s using a Langevin thermostat and periodic boundary conditions at constant pressure. To describe long-range electrostatic effects, the particle mesh Ewald technique (PME) and periodic boundary conditions (PBC) were used, with a weak coupling algorithm used to control the temperature from an external bath. The SHAKE algorithm was used to restrain the lengths of hydrogen bonding [35]. The Langevin coupling integration algorithm was used to keep the temperature constant. To solve Newton’s equations, a time step of 2 fs was chosen, and the trajectory data were gathered every 1 ps for the subsequent investigation. All MD trajectory studies were performed using the CPPTRAJ module in AmberTools20, and visual examination was carried out using Visual Molecular Dynamics software [36].

### 2.11. MMPB/GBSA Analysis

Compounds binding free energy towards receptor was measured in order to confirm the compounds’ binding stability. The molecular mechanics generalized Poisson Boltzmann surface area (MMPBSA) approach was used to achieve this. This approach is a widely used, dependable, and strong analytical technique [37,38,39,40]. The Amber tool 20 MMPBSA script (py) was used to assess binding free energy of selected MD snapshots. 

In short, the binding free energy was computed using the equation below:

ΔGbinding = ΔEMolecularMechanics + ΔGsolvation − TΔGΔGbinding = ΔEMolecularMechanics + ΔGsolvation − TΔS(entropy)(1)

## 3. Results

### 3.1. Core Proteome Retrieval

Complete proteome of four strains of *Streptococcus pyogenes*—*S. pyogenes* serotype M1 (strain ATCC 700294/SF370, accession no: UP000000750), *S. pyogenes* M1 476 (accession no: UP000005248), *S. pyogenes A20* (accession no: UP000001267), and *Streptococcus pyogenes* serotype M3 (strain ATCC BAA-595/MGAS315; accession no: UP000000564)—were downloaded in the FASTA format from UniProt. These proteomes were subjected to OrthoFinder for core proteome retrieval. OrthoFinder server predicted 1324 core proteins.

### 3.2. Identification of Drug Targets

Essential proteins are involved in the synthesis of antibacterial compounds; hence, they are promising targets for drug development [41]. The Geptop 2.0 server predicted 302 essential proteins out of the 1324 core proteins. Proteins involved in multiple common cellular processes in *H. sapiens* and bacteria evolved as homologs over time. To be considered as an effective drug target, the proteins must be important for the pathogen’s survival within the host’s body, while also being non-homologous to host proteins. This requirement is necessary to avoid drug–host protein cross-binding, which would increase the likelihood of pharmacological side effects [42,43]. As a result, BLASTp was run against *H. sapiens* for all 302 essential proteins. The results revealed 145 non-homologous protein sequences with less than 30% identity. Protein function can be assessed quickly and inexpensively by predicting its subcellular localization. It was also discovered that proteins can be found in a variety of locations, making localization an important consideration when designing any therapeutic agent. As drug targets, cytoplasmic proteins are preferred because membrane proteins are more challenging to analyze and purify [43,44]. According to the results of the BUSCA server, 46 proteins were classified as cytoplasmic proteins.

The metabolic pathways where these 46 cytoplasmic proteins play a role were analyzed, and it was discovered that they were engaged in 19 pathways. A comparison of the metabolic pathways of *S. pyogenes* and *H. sapiens* was carried out to choose drug candidates engaged in pathogen-specific pathways. When the pathways of *S. pyogenes* and *H. sapiens* were compared, 6 were determined to be pathogen-specific, while the other 13 were shown to be shared by host and the pathogen. These 6 unique pathways were discovered to involve a total of 10 *S. pyogenes* cytoplasmic proteins. The KEGG database was used to further analyze these 10 proteins. Two of the ten proteins were discovered to be involved in unique pathways, whereas the other eight were found to be related with pathways that were found in the pathogen and the host and were thus ruled out of further investigation (Table 1).

Two proteins, i.e., chromosomal replication initiator protein DnaA and two-component response regulator (TCR), were identified as novel drug targets. These two proteins were linked to a single metabolic pathway, i.e., a two-component system. Proteins present in unique metabolic pathways can be considered pathogen-specific and could be used as vaccine and drug targets [45].

### 3.3. Structure Prediction

**Figure 1:**
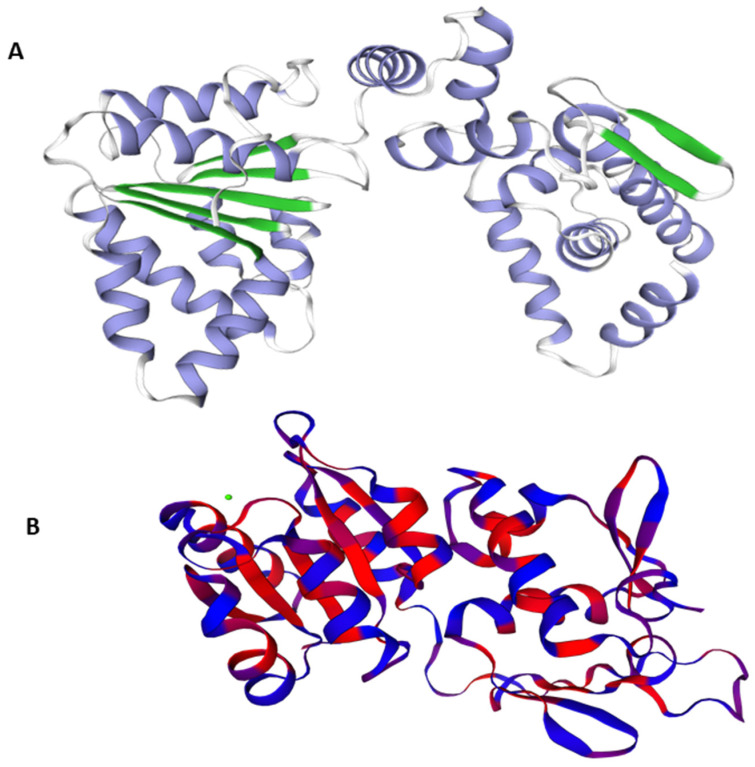
3D structures of the target proteins DNaA (**A**) and TCR (**B**).

The best Predicted 3D crystal structure for both proteins were chosen from Swiss Model on the basis of QMEAN and GMQE values as shown in Figure 1. 

A model’s confidence level is determined by GMQE (global model quality estimation), which needs to take into account the template, coverage, and organization of the target. For calculating quality, it integrates target–template alignment properties with template search. The better the model’s quality, the higher the GMQE score. It is typically estimated between 0 to 1. The GMQE was 0.73 for DNaA and 0.48 for TCR, while the Q mean was −0.90 for DNaA and −2.80 for TCR, depicting the high quality of the structures.

### 3.4. Model Evaluation

Refined drug targets were further evaluated to predict the quality of the protein structures. Multiple methods were employed for validation of the 3D models. Firstly, PROCHECK server was used for the structural quality assessment of the modeled structure. Predicted model evaluation of the DNaA protein showed that 88.4% of residues were in favored regions, while TCR protein showed 89.6% of residues in favored regions. Conclusively, evaluation of the 3D protein models demonstrated that nearly 90% of residues were in the favored and allowed regions, thus confirming that both predicted models are of high-quality. VERIFY 3D predicted that in the DNaA model, 74.71% of the residues had averaged 3D–1D score ≥ 0.2, while in the case of the TCR model, 85.47% of the residues had averaged 3D–1D score ≥ 0.2, thereby verifying the model in the context of structure–sequence compatibility. ERRAT, a so-called quality factor, predicted the quality score of the DNaA model as 90.0602, while for the TCR model, the predicted quality factor was 92.4779. The higher the score, the more significant the quality of the 3D model. These findings demonstrate that the refined model is of high quality. The ProSA-web server was employed to double-check the quality of the 3D models. The Z-scores, a parametric quantity representing the overall quality of the model, were −8.48 and −7.86 for DNaA and TCR proteins, respectively. Table 2 summarizes the findings of these four programs, indicating the high quality of the models. 

### 3.5. Molecular Docking Analysis

**Figure 2:**
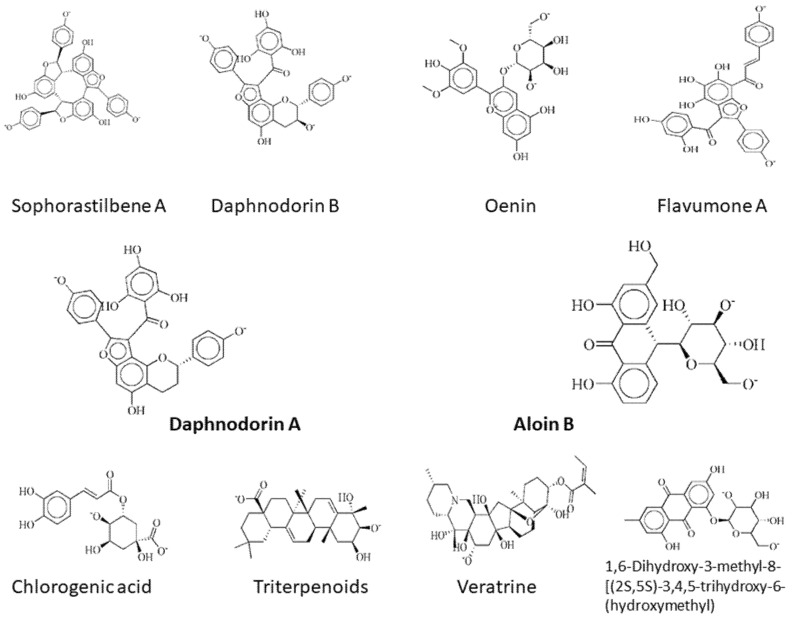
2d structures of the top drug candidates. First five compounds, sophorastilbene A, daphnodorin B, oenin, flavumone A and daphnodorin A, are considered as drug target against DNaA protein, while the remaining compounds, aloin B, chlorogenic acid, triterpenoids, veratrine, and 1,6-dihydroxy-3-methyl-8-[(2S,5S)-3,4,5-trihydroxy-6-(hydroxymethyl)oxan-2-yl]oxyanthracene-9,10-dione, were considered as drug candidates for TCR protein.

Lys115, Tyr116, Asn120, Phe121, Ile122, Glu126, Asn127, Gly153, Lys291, Asn290, Lys2, Asp47, Leu161, Arg118, and His72 are the important residues and the active binding regions of the DNaA and TCR proteins that were predominantly engaged in ligand–protein interactions. All those binding pockets were selected by the site finder tool present in the molecular operating environment. Top 10 inhibitors, sophorastilbene A, daphnodorin B, oenin, flavumone A, daphnodorin A, aloin B, chlorogenic acid, triterpenoids, veratrine and 1,6-dihydroxy-3-methyl-8-[(2S,5S)-3,4,5-trihydroxy-6-(hydroxymethyl)oxan-2-yl]oxyanthracene-9,10-dione, were screened out of 1000 showing good docking score along with RMSD values for both target proteins, as shown in Figure 2. 

With DNaA as a target, sophorastilbene A inhibitor showed best binding score. The aloin B inhibitor with the TCR protein as a target showed top docking score with well interacting residues Arg118 and His72. The same approach was employed to carry out the docking analysis of the remaining inhibitors after the docking process was confirmed. For both target proteins, the docking score and the interactions between active sites and the ligands bound are given in the Table 3.

**Figure 3:**
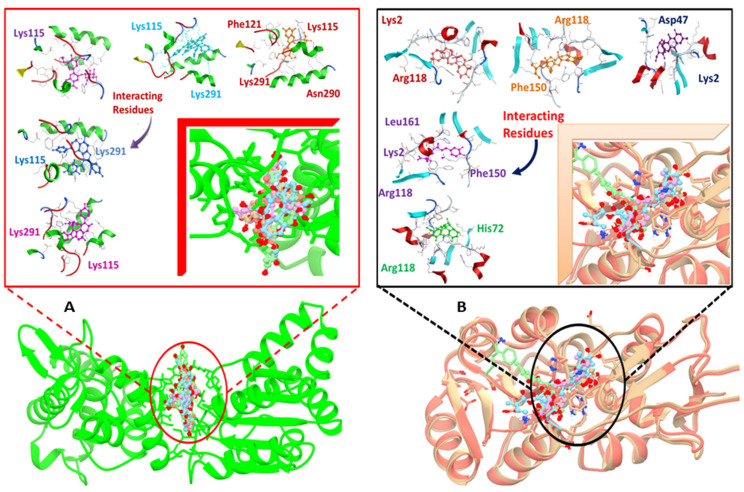
Docked complexes indicating, in detail, the interacting residues of the DNaA (**A**) and TCR (**B**) target proteins.

It involved a variety of amino acid residues and interactions for phytochemicals to bind to the active pocket of the target proteins. Lys291, Lys115, Arg118, Lys2, and Phe150 in hydrogen bonding and pi-stacking interactions were the primary residues involved in building the contacts between the top hit ligand conformations and the binding pocket of the target proteins, as shown in Figure 3A,B. 

Those interactions were predicted using the LigX tool as shown in the Supplementary Figures S1–S10.

### 3.6. Druglikeness Prediction

Computational scanning of the physicochemical properties of the best-docked ligands for both targets was performed using the Lipinski’s rule of five (RO5) to evaluate their drug-like features. According to this rule, the molecular weight is less than or equal to 500 g/mol, number of hydrogen bond donors less than or equal to 5, number of hydrogen bond acceptors less than or equal to 10, and miLog *p* value less than equal to five. A drug candidate with one rule violation is acceptable. The top hit phytochemicals and the reference compound’s projected druglikeness properties are shown in Table 4. All the reported ligands had excellent druglike characteristics.

### 3.7. ADMET Profiling

The Swiss ADME (UNIL University of Lausanne, Lausanne, Switzerland) and admetSAR (East China University of Science and Technology, Shanghai, China) tools were used to predict different types of pharmacokinetic properties. Pharmacokinetic factors can be used to forecast the top drug candidate molecules’ absorption, distribution, metabolism, elimination (ADME), and toxicity. For both targets, the ADMET characteristics of derived phytochemicals are shown in Table 5. Drug development of many drugs does not include this process because of poor pharmacokinetic properties and toxicity [46]. Identification of active lead compounds depends upon the high-performance and fast ADMET profiling assays in early drug discovery [47,48]. The ADMET profiling showed that there was no side effect of absorption of all the candidate compounds. The associated ADMET properties of potential compounds for different models, such as P-glycoprotein substrates, BBB penetration, and gastrointestinal absorption, showed positive results that strongly support the compounds’ suitability as drug candidates.

### 3.8. MD Simulation

**Figure 4:**
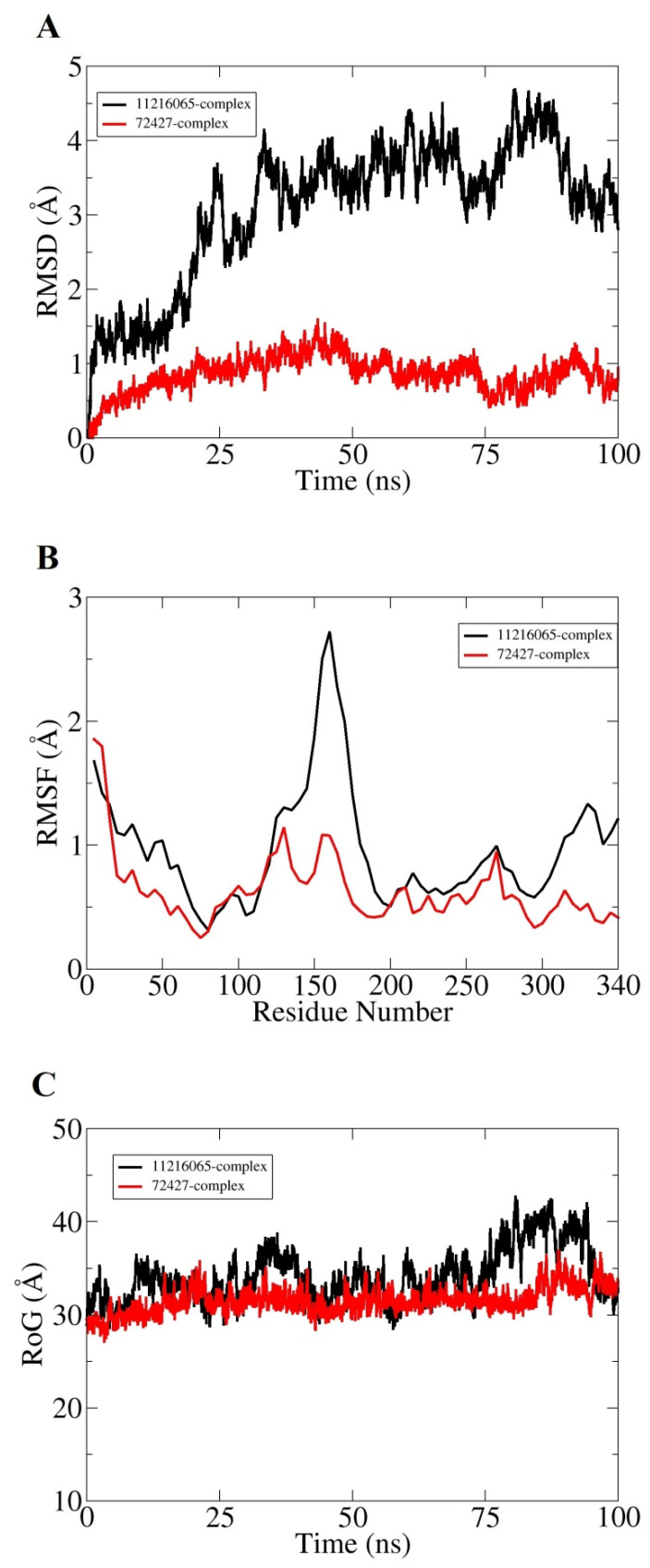
Statistical analysis based on molecular dynamics simulations to assess the intermolecular stability and dynamics of the two complexes of the DNaA protein. (**A**) part is indicating the stability of the compounds by Root Mean Square Deviation, (**B**) part is indicating the Root mean square fluctuation plots for top two hits and (**C**) part is indicating the Radius of Gyration values for the top hits over the time period of 100 ns.

**Figure 5:**
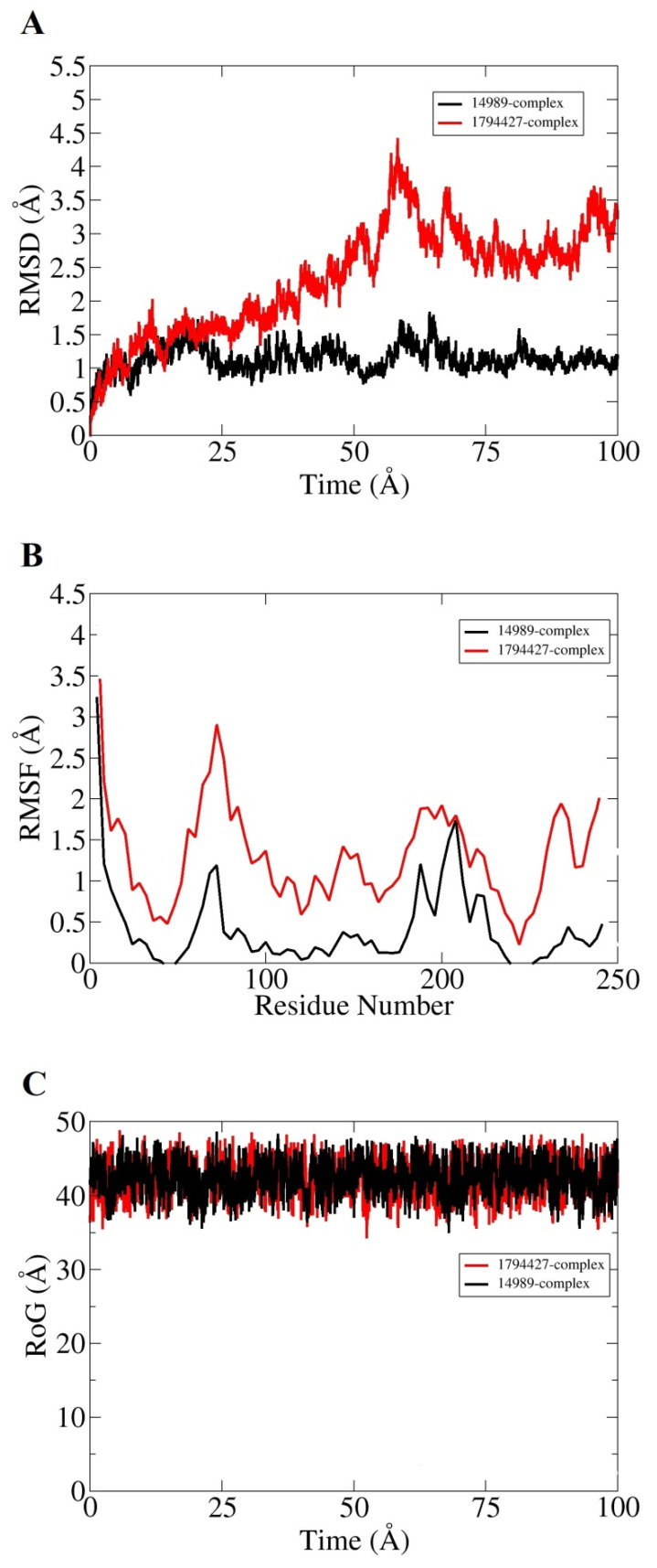
Statistical analysis based on molecular dynamics simulations to assess the intermolecular stability and dynamics of the two complexes of the TCR target proteins. (**A**) part is indicating Root Mean Saquare Deviation for both top compounds similarly; (**B**) part is indicating Root Mean Square Fluctuation; (**C**) part is indicating graph values for Radius of Gyration both hits can be presented in different colors as mention above.

Molecular dynamic simulation for 100 ns was performed to understand dynamics of both targets in the presence of screened hits. Statistical parameters RMSD, RMSF, and RoG were studied for docked complexes to confirm their structural stability. The root mean square deviations (RMSD) were investigated first based on carbon alpha atoms. Deviation in the RMSD plot is an indication of structural variations relative to the initial docked complex inter-molecular conformation [49]. Uniform RMSD plot implies structure equilibrium of the system and more inter-molecular strength as the simulation time proceeds. The DNaA–72427 complex remained stable during the first 50 ns of simulation time; after that, it showed lower RMSD deviation of 0.25 Å and got more stability until the simulation end. The second complex of DNaA (with 11216065) showed stability up to 20 ns; after that, it showed deviations, though minor (of 1 Å), and achieved stability towards the end, as shown in Figure 4A. The TCR–14989 complex showed stability throughout the time frame up to 100 ns, and RMSD reached the maximum of 1 Å. The second complex (with 1794427) showed steady RMSD with deviation at 60 ns, and after that, it showed stability, as indicated in Figure 5A. From the RMSD interpretation, it can be easily concluded that DNaA–72427 is showing more intermolecular stability in term of chemical interactions and conformation thus 72427 can be regarded as high affinity drug molecule for DNaA. On the other side, 14989-TCR complex is more stable in term of binding affinity compared to 1794427-TCR complex. The same trend was noticed for both proteins in remaining simulation analysis. 

Root mean square fluctuations (RMSF) were calculated next for the simulated complexes. RMSF analysis aids in identifying flexible residues of the targeted proteins and understanding how these fluctuations are affecting complex stability [50]. Graphs of the DNaA target indicate that its complex with 72427 showed minor fluctuations, while its complex with 11216065 showed a deviation of 1.5 Å at one point, as shown in the Figure 4B. The RoG plot trajectories of the DNaA target for the first complex (with 72427) showed a good stability, while the second complex (with 11216065) showed minor deviations indicated in Figure 4C. However, the RMSF plot of the target TCR–14989 complex showed stability at 0.5 Å, while the TCR–1794427 complex showed stability between 1 Å and 1.5 Å, as shown in the Figure 5B. Radius of gyration (RoG) is a parameter reflecting the system’s compactness during simulation time. Lower RoG value refers to a highly compact system and is an indication of system stability. The RoG values for the target TCR showed a good stability for both complexes throughout the time period of 100 ns, as shown in the Figure 5C. All these analyses indicated the targeted proteins were in good dynamics stability in the presence of the virtually screened compounds. The complex intermolecular stability also demonstrated the best fitting between the proteins and the compounds, leading to strong association and, ultimately, stable formation of complexes.

### 3.9. Binding Free Energy Calculations

The calculation of the binding free energy of docked complexes was carried out using the MMGBSA method, as represented in the Table 6. The data demonstrated that the molecular recognition was dominated by gas phase energy, in particular, by electrostatic energy and van der Waals energy. The polar solvation energy seemed to play a smaller role in molecules’ interactions with the targeted proteins. The non-polar energy had a marginally significant contribution in complex formation. The total binding energies of both complexes for target TCR were −21.14 kcal mol^−1^ and −21.18 kcal mol^−1^, and similarly, for target DNaA, it was −16.24 kcal mol^−1^ and −21.83 kcal mol^−1^, respectively.

## 4. Discussion

*Streptococcus pyogenes* is a common human pathogen that produces a spectrum of disorders, from minor issues, such as pharyngitis and impetigo, to more serious infections, such as necrotizing fasciitis, sepsis, and toxic shock syndrome [51]. To fight these life-threatening scenarios, medications against *S. pyogenes* must be developed as soon as possible. In silico core proteomics and molecular docking approaches were used to search for therapeutic candidates against *S. pyogenes* in our research work. These approaches are used to identify targets in pathogenic organisms based on the identification of essential proteins. In computer-based drug design techniques, identifying therapeutic targets is a vital step [52]. Recent breakthroughs in the fields of bioinformatics and computational biology have resulted in a number of approaches to drug design and in silico analysis, lowering the time and cost of drug development through trial and error [53]. 

The core proteome of *S. pyogenes* strains, which contained 1324 proteins, was subjected to the Geptop 2.0 server for essential proteins prediction. Essential proteins are required for bacterial survival. Bacteria cannot survive if these essential proteins are mutated or degraded. We can kill bacteria and cure disease by targeting these proteins. Essential proteins are the preferred targets for antibacterial drug and vaccine development. Hence, 302 essential proteins were identified among the core proteins. This method was used by Sakharkar et al. to identify 306 essential genes in *P. aeruginosa*, Chong et al. to identify 312 essential proteins in *Burkholderia pseudomallei*, and Shiragannavar et al. to identify 807 essential proteins in *Eubacterium nodatum* [49]. These genes may be similar to those found in humans. As a result, targeting such genes may prove fatal and disrupt human metabolism. Selection of non-homologous proteins not found in *Homo sapiens* can reduce the risk of adverse events and cross-reactivity [54]. We screened 145 non-homologous proteins to avoid such unfavorable situations and toxicity. Targeting and developing inhibitors against non-homologous sequences for the development of new drugs may be the best strategy.

A comparison of human and pathogen metabolic pathways using the KEGG database revealed that 6 pathways are unique to pathogens and 13 are shared by both pathogens and hosts. A total of 10 *S. pyogenes* essential proteins were involved in these 6 pathways. The results of pathogen-specific pathway identification are consistent with those reported by Goyal et al., Amineni et al., and Shahid et al. for *A. baumannii, L. interrogans*, and *S. saprophyticus*, respectively [13,55]. Eight out of the ten proteins were found to be involved in *H. sapiens* and *S. pyogenes* pathways. Two proteins, i.e., chromosomal replication initiator protein DnaA and two-component response regulator (TCR), were found to be involved in unique metabolic pathway, i.e., a two-component system, and identified as novel drug targets. Several other studies have also reported DnaA [56,57] as a drug target.

Three-dimensional structures of the target proteins were modeled using the Swiss model. Prediction of the 3D structure is very useful in studying protein dynamics, functions, ligand interactions, and other protein components, and it provides a lot of information about the spatial structure of important protein components [58,59]. The Ramachandran plot analysis revealed that the majority of residues were in acceptable, favored areas, with very few residues in the disallowed region, and the model’s overall quality was satisfactory. Values of other evaluation tool indicated a high quality of our models.

To select the compounds having the best residue interaction with the target protein, the molecular docking approach was used. Out of 1000 docked molecules, sophorastilbene A, daphnodorin B, oenin, flavumone A and daphnodorin A were chosen for their low RMSD 3 score and their large number of residues interacting with the target protein DNaA, and aloin B, chlorogenic acid, triterpenoids, veratrine and 1,6-dihydroxy-3-methyl-8-[(2S,5S)-3,4,5-trihydroxy-6-(hydroxymethyl)oxan-2-yl]oxyanthracene-9,10-dione were chosen for the TCR protein. The lowest binding energies of these phytochemicals ranged from about −21.31 kcal/mol to −16.00 kcal/mol for the DNaA protein and −18.02 kcal/mol to −16.84 kcal/mol for the TCR protein. The chemical profile and drug likelihood of these 10 compounds were evaluated using the Lipinski’s rule of five. Afterwards, the compounds were examined for BBB penetration and HIA (human intestinal absorption) and subjected to the AMES monitoring. ADMET characteristics are a key predictor of a drug candidate’s behavior, toxicity, and fate in the human body. They indicate the candidate’s likelihood of intestinal absorption, metabolism, crossing the blood–brain barrier, subcellular localization, and, most critically, the level of harm it can cause in the body [60]. CYP2A6, CYP1A2, CYP2C9, CYP2D6, CYP2C19, CYP3A4, and CYP2E1 are the isoforms of the cytochrome P450 superfamily that are involved in drug metabolism and hepatic clearance [61]. As a result, blocking the cytochrome P450 isoforms can produce a drug–drug interaction that prevents the metabolism of concomitant medications, leading to hazardous levels of accumulation [62]. 

The ADMET profile of these chemicals reveals that they have no negative effects on absorption. Furthermore, when compared with the AMES test, none of the chemicals were hazardous or mutagenic. The 10 hit compounds produced following the virtual screening were subjected to several toxicity modules. No chemical was determined to be cytotoxic, hepatotoxic, or mutagenic in the course of the toxicity evaluation. Our research revealed 10 drug-leading inhibitors that, by successfully targeting and blocking apoptosis, could be therapeutic inhibitors of DnaA and TCR.

Molecular dynamics (MD) simulations and free energy calculations were performed on the best docked complexes with two inhibitors per protein because these ligands demonstrated great binding affinity, as evidenced by a high dock score and a good molecular interaction network.

Previously, using in silico comparative studies, 18 enzymes were identified as drug targets against *S. pyogenes*. These enzymes showed major drug-target properties including their involvement in pathogen metabolic pathways, non-homology to the human host, and appropriate size. Three enzymes (DNA polymerase III subunit beta, [acyl-carrier-protein] S-malonyltransferase, and dihydropteroate synthase) were reported as novel drug targets against the pathogen [9]. However, in this study, only two drug targets were identified on the basis of unique metabolic pathway. Drug targets and drug-like compounds prioritized in this study could be useful in developing new strategies to combat *S. pyogenes* drug resistance.

## 5. Conclusions

The rapid emergence of antimicrobial resistance among Gram-positive bacteria has prompted the need to investigate novel drug targets that could aid in the development of new antimicrobial agents. The current study identified two novel targets in *S. pyogenes.* Because both are involved in pathogen-specific metabolic pathways and have successfully been targeted in other microbes, the current study explored developing drugs against them. Thus, this study represents a significant advance in the design of new, potent compounds against *S. pyogenes*. These targets should be tested experimentally in future studies to determine their role in *S. pyogenes* survival and virulence.

## Figures and Tables

**Table 1:** Ten cytoplasmic proteins presented in unique metabolic pathways.

Name	Common Pathways	Unique Pathways
Glucose-6-phosphate isomerase	Metabolic pathways Glycolysis Carbon metabolism Pentose phosphate pathway Amino sugar and nucleotide sugar metabolism Starch and sucrose metabolism	Biosynthesis of secondary metabolites Microbial metabolism in diverse environments
UDP-N-acetylenolpyruvoylglucosamine reductase	Metabolic pathways Amino sugar and nucleotide sugar metabolism	Peptidoglycan biosynthesis
Riboflavin biosynthesis protein	Biosynthesis of cofactors Metabolic pathways Riboflavin metabolism	Biosynthesis of secondary metabolites
Alanine racemase	Metabolic pathways	d-Alanine metabolism Vancomycin resistance
Chromosomal replication initiator protein DnaA		Two-component system
Two-component response regulator		Two-component system
Phosphate acyltransferase	Glycerolipid metabolism Metabolic pathways	Biosynthesis of secondary metabolites
Fructose-bisphosphate aldolase	Metabolic pathways Glycolysis Carbon metabolism Biosynthesis of amino acids Fructose and mannose metabolism Pentose phosphate pathway Methane metabolism	Biosynthesis of secondary metabolites Microbial metabolism in diverse environments
UDP-N-acetylmuramoyl-tripeptide—D-alanyl- D-alanine ligase	Metabolic pathways	Vancomycin resistance Peptidoglycan biosynthesis
Acetyl-coenzyme A carboxylase carboxyl transferase subunit alpha	Metabolic pathways	Biosynthesis of secondary metabolites

**Table 2:** Evaluation of 3D model using PROCHECK, VERIFY3D, ERRAT, and ProSA-Web servers.

Target Proteins	Ramachandran Plot Statistics (%)				Verify 3D	ERRAT	ProSA
	Core	Allowed	General	Disallowed	Compatibility Score (%)	Quality Factor	z-Score
Chromosomal replication initiator protein DnaA	88.4%	10.0%	1.6%	0.0%	74.71%	90.0602	−8.48
Two-component response regulator	89.6%	9.5%	0.5%	0.5%	85.47%	92.4779	−7.86

**Table 3:** The top 10 bioactive phytochemicals interactions along with the docking analysis results.

Target Proteins	Compound ID’s	Compounds Name	Docking Score (kcal/mol)	RMSD	Interacting Residues
DNaA Protein	11216065	Sophorastilbene A	−21.31	3.61	Phe121 **Lys115** **Lys291** Asn290
	72427	Daphnodorin B	−20.77	1.70	**Lys115** **Lys291**
	443652	Oenin	−20.18	1.96	**Lys115**
	12096478	Flavumone A	−20.06	2.32	**Lys115** **Lys291**
	72426	Daphnodorin A	−16.00	3.26	**Lys115** **Lys291**
TCR protein	14989	Aloin B	−18.02	1.55	**Arg118** His72
	1794427	Chlorogenic acid	−17.47	1.23	**Lys2** **Arg118** **Phe150** Leu161
	71597391	Triterpenoids	−17.47	1.42	**Lys2** **Arg118**
	5380394	Veratrine	−16.96	2.22	**Arg118** **Phe150**
	118855584	1,6-Dihydroxy-3-methyl-8-[(2S,5S)-3,4,5-trihydroxy-6-(hydroxymethyl)oxan-2-yl]oxyanthracene-9,10-dione	−16.84	1.51	Asp47 **Lys2**

**RMSD**: Root Mean Square Deviation; Bold Interacting Residues indicate that all the compounds are hitting the common targets.

**Table 4:** Druglike characteristics of top hits.

Target Proteins	Compounds	Molecular Weight (g/mol)	Number of HBA (nON)	Number of HBD (nOHNH)	mi-LogP
**DNaA**	11216065	673.65	9	3	4.36
	72427	539.47	10	4	2.24
	443652	491.43	12	5	−2.39
	12096478	538.46	10	5	2.29
	72426	524.48	9	4	3.25
**TCR**	14989	416.38	9	5	−3.04
	1794427	352.30	9	4	−3.24
	71597391	470.65	5	2	1.70
	5380394	590.73	10	5	−0.54
	118855584	430.37	10	4	−1.82

**HBD:** Hydrogen Bond Donors; **HBA:** Hydrogen Bond Acceptor.

**Table 5:** Pharmacokinetic properties of the top predicted drug candidates for the DNaA and TCR proteins.

Standard Parameters	Target									
	DNaA					TCR				
	11216065	72427	443652	12096478	72426	14989	1794427	71597391	5380394	118855584
**Absorption**										
Aqueous solubility (LogS)	−3.3170	−3.2562	−2.7564	−3.0485	−2.9143	−2.3269	−2.5951	−3.9108	−2.4829	−2.8081
Human Intestinal Absorption	0.9727	+ 0.9394	−0.9165	+0.9300	+0.8623	+0.7201	−0.8658	+0.7320	−0.7652	−0.8845
Blood Brain Barrier	+0.8635	+0.7154	−0.8897	+0.6248	+0.7183	+0.5432	+0.5612	+0.7302	−0.8131	−0.6852
Caco-2 permeability	−0.6888	−0.8292	−0.4982	−0.0172	0.6303	0.2248	−0.5040	1.1647	0.2244	−0.4438
**Distribution**										
P-gp Substrate	Non-Substrate	Non-Substrate	Substrate	Substrate	Substrate	Substrate	Substrate	Substrate	Substrate	Substrate
P-gp Inhibitor	Non-Inhibitor	Non-Inhibitor	Non-Inhibitor	Non-Inhibitor	Non-Inhibitor	Non-Inhibitor	Non-Inhibitor	Non-Inhibitor	Inhibitor	Non-Inhibitor
**Metabolism**										
CYP450 2D6 Substrate	x	x	x	X	x	x	x	x	x	x
CYP450 3A4 Substrate	x	x	√	X	x	x	x	√	√	x
CYP450 1A2 Inhibitor	√	√	X	√	√	x	x	x	x	x
CYP450 2C9 Inhibitor	√	√	X	√	√	x	x	x	x	x
CYP450 2D6 Inhibitor	x	x	X	X	x	x	x	x	x	x
CYP450 2C19 Inhibitor	√	√	X	X	√	x	x	x	x	x
CYP450 3A4 Inhibitor	x	√	X	X	√	x	x	x	x	x
**Toxicity**										
*Salmonella typhimurium* reverse mutation assay AMES Test	Non-AMES Toxic	Non-AMES Toxic	Non-AMES Toxic	Non-AMES Toxic	Non-AMES Toxic	AMES Toxic	Non-AMES Toxic	Non-AMES Toxic	Non-AMES Toxic	AMES Toxic
Human Ether-à-go-go-Related Gene (hERG) Inhibition	Weak inhibitors	Weak inhibitors	Weak inhibitors	Weak inhibitors	Weak inhibitors	Weak inhibitor	Weak inhibitor	Weak inhibitor	Weak inhibitor	Weak inhibitor
Carcinogens	Non-Carcinogens	Non-Carcinogens	Non-Carcinogens	Non-Carcinogens	Non-Carcinogens	Non-Carcinogens	Non-Carcinogens	Non-Carcinogens	Non-Carcinogens	Non-Carcinogens
Rat Acute Toxicity (LD50, mol/kg)	2.4083	2.5846	−2.7564	2.5248	3.0847	2.5732	2.6020	2.8611	3.3693	2.9432

HIA: human intestinal absorption (%), Caco-2: colorectal carcinoma; BBB: blood-brain barrier; CYP450: cytochrome P450; P-gp: P-glycoprotein; hERG: human ether-à-go-go related gene channel inhibition. Solubility level: insoluble, less than −10; poorly, between −10 and −6; moderately, between −6 and −4; soluble, between −4 and −2; very, between−2 and 0; highly, more than 0. HIA level: 0–20%, poorly absorbed; 20–70% moderately absorbed; 70–100%, well absorbed. BBB level (concentration in brain and concentration in blood): absorption to central nervous system less than 0.1, low; between 0.1 and 2.0, moderate; more than 2.0, high.

**Table 6:** Binding energies of the target proteins. All values are given in kcal/mol.

Energy Parameter	TCR		DNaA	
	14989-Complex	1794427-Complex	72427-Complex	11216065-Complex
**MM-GBSA**				
VDWAALS	−22.23	−21.65	−18.16	−22.46
EEL	−10.11	−9.10	−11.21	−10.58
EGB	13.20	12.11	16.58	15.21
ESURF	−2.00	−2.54	−3.39	−4.00
Delta G gas	−32.34	−30.75	−29.37	−33.04
Delta G solv	11.20	9.57	13.13	11.21
Delta Total	−21.14	−21.18	−16.24	−21.83
**MM-PBSA**				
VDWAALS	−22.23	−21.65	−18.16	−22.46
EEL	−10.11	−9.10	−11.21	−10.58
EPB	10.28	6.28	12.07	9.41
ENPOLAR	−5.00	−3.96	−2.98	−3.51
Delta G gas	−32.34	−30.75	−29.37	−33.04
Delta G solv	5.28	2.32	9.09	5.9
Delta Total	−27.06	−28.43	−20.28	−27.14

## Data Availability

Not applicable.

## Supplementary Materials

The following are available online at https://www.mdpi.com/article/10.3390/ijerph182111355/s1, Figures S1–S5: Two-dimensional interaction images of (S1) flavumone A with DNaA target protein, (S2) daphnodorin A with DNaA target protein, (S3) oenin with DNaA target protein, (S4) sophorastilbene A with DNaA target protein, and (S5) daphnodorin B with DNaA target protein. Figures S6–S10: Two-dimensional interaction images of (S6) Aloin B with TCR target protein target protein, (S7) Chlorogenic acid with TCR target protein, (S8) Triterpenoids with TCR target protein, (S9) Veratrine with TCR target protein, (S10) 1,6-dihydroxy-3-methyl-8-[(2S,5S)-3,4,5-trihydroxy-6-(hydroxymethyl)oxan-2-yl]oxyanthracene-9,10-dione with TCR target protein.
