# Supplementary material for: In Silico Core Proteomics and Molecular Docking Approaches for the Identification of Novel Inhibitors against Streptococcus pyogenes
Source: Int J Environ Res Public Health. 2021 Oct 28;18(21):11355. doi: 10.3390/ijerph182111355 (PMC8582943; doi:10.3390/ijerph182111355)
Supplement: Supplementary file 1 [file ijerph-18-11355-s001.zip › ijerph-1374913-supplementary.pdf]

S-1

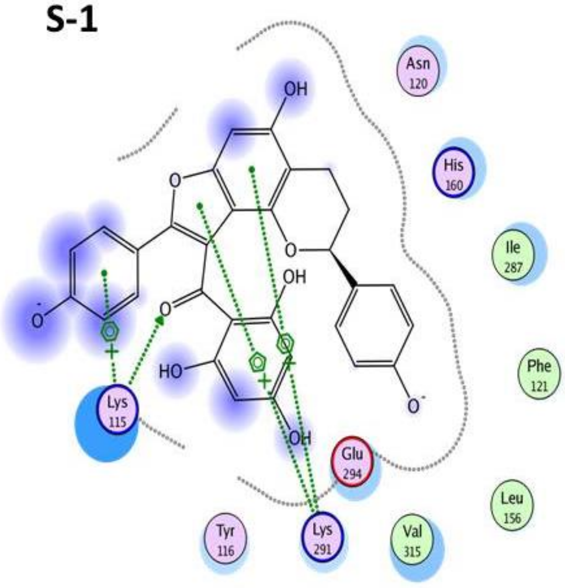

S-2

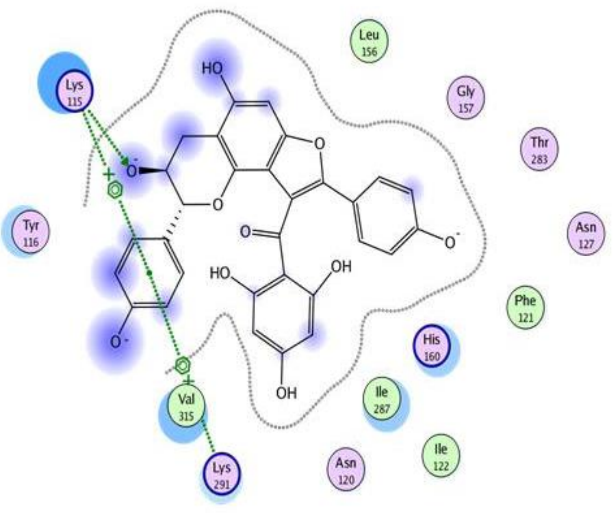

Figure (S1-S2): 2D interaction images of **S1**) Flavumone A with DNaA target protein **S2**) Daphnodorin A with DNaA target protein

S-3

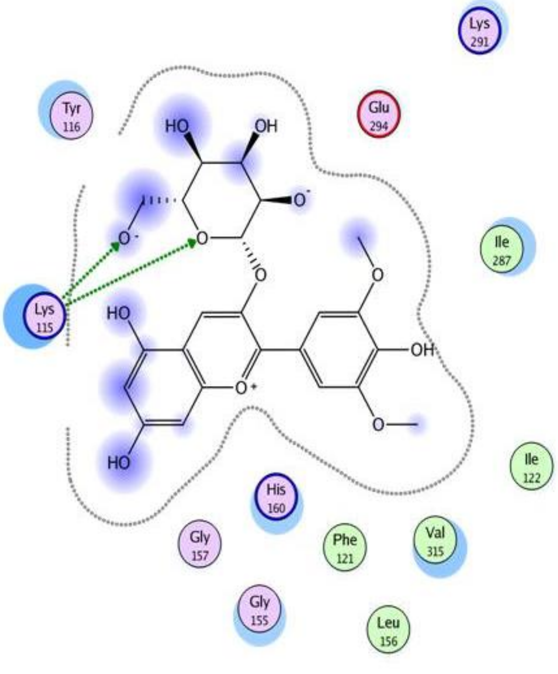

S-4

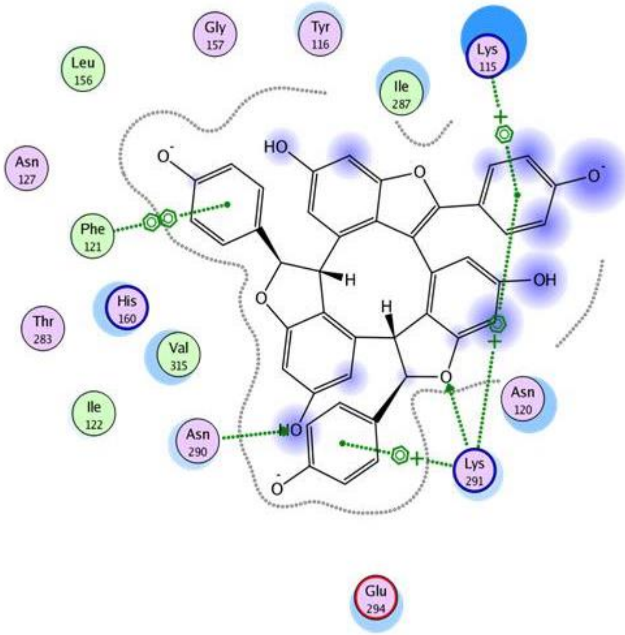

Figure (S3-S4): 2D interaction images of **S3)** Oenin with DNaA target protein **S4)** Sophorastilbene A with DNaA target protein

S-5

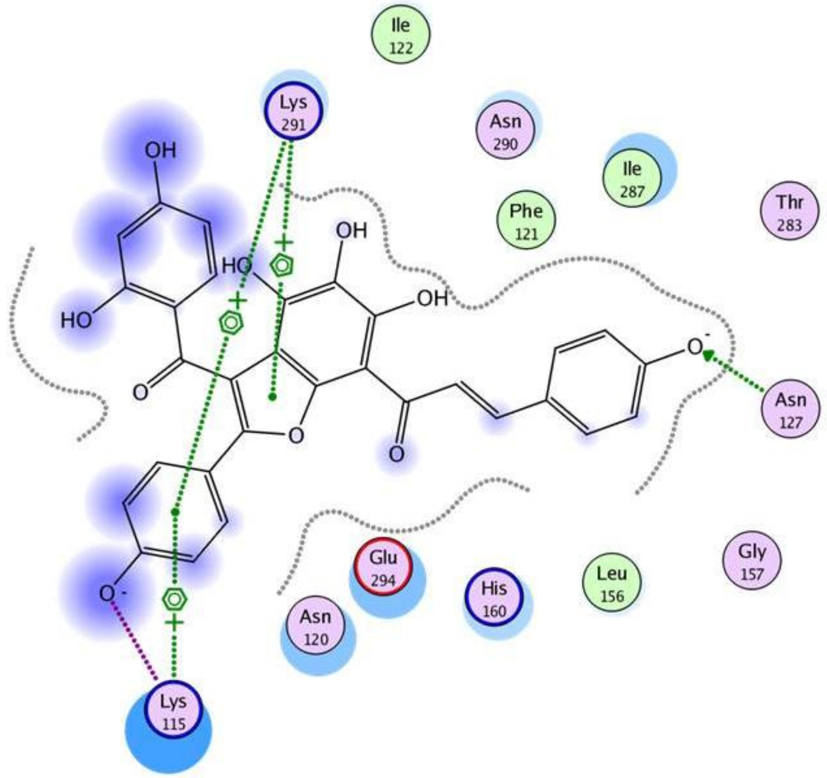

Figure (S5): 2D interaction images of **S5)** Daphnodorin B with DNaA target protein.

S-6

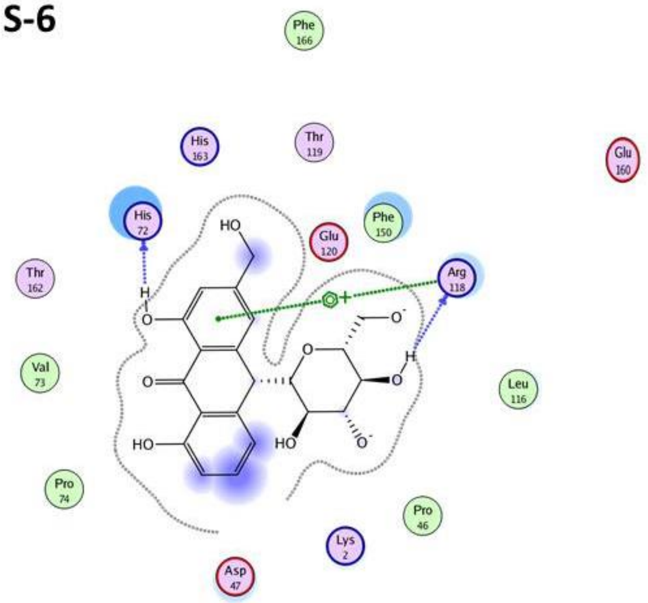

S-7

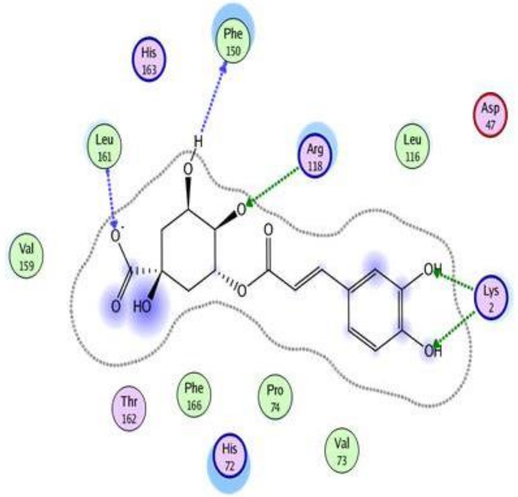

Figure (S6-S7): 2D interaction images of **S6)** Aloin B with TCR target protein and **S7)** Chlorogenic acid with TCR target protein

S-8

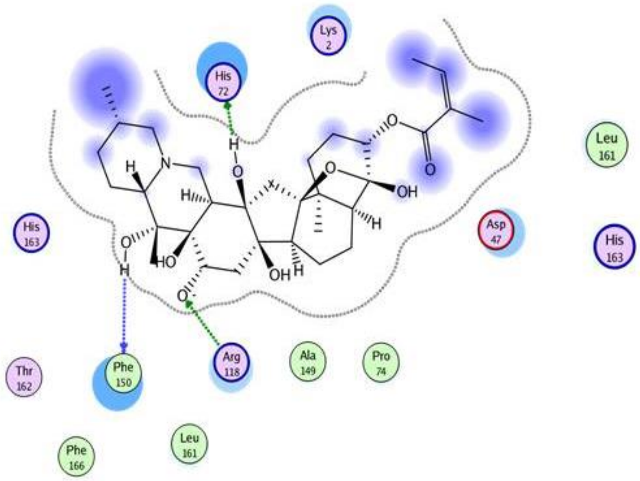

S-9

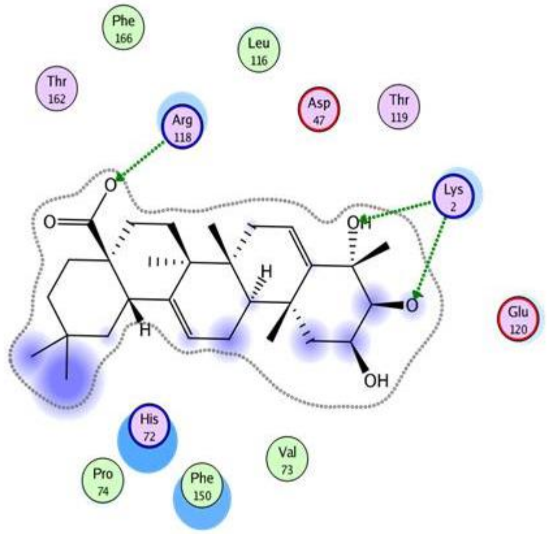

Figure (S8-S9): 2D interaction images of **S8)** Triterpenoids with TCR target protein **S9)** Veratrine with TCR target protein)

S-10

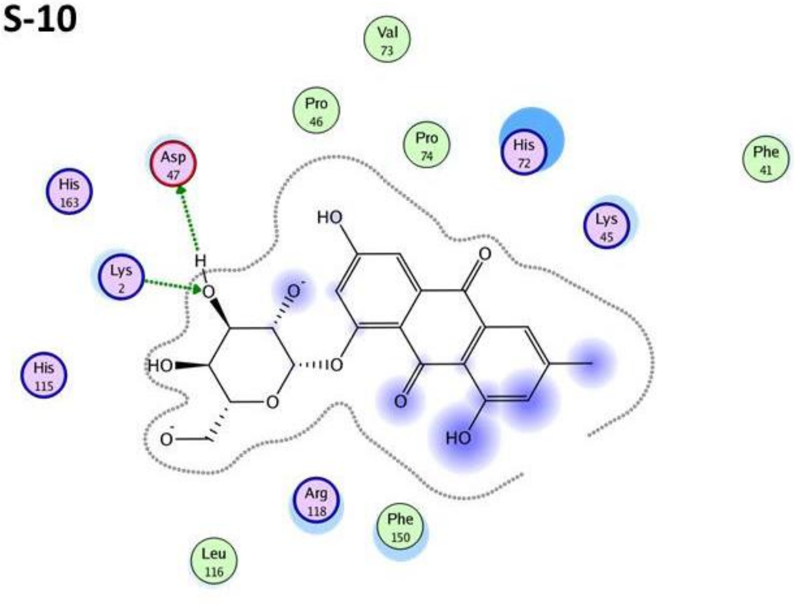

Figure (S10): 2D interaction images of **S10)** 1,6-Dihydroxy-3-methyl-8-[(2S,5S)-3,4,5-trihydroxy-6-(hydroxymethyl)oxan-2-yl]oxyanthracene-9,10-dione with TCR target protein
